# Cytotoxic and antiangiogenic activity of AW464 (NSC 706704), a novel thioredoxin inhibitor: an *in vitro* study

**DOI:** 10.1038/sj.bjc.6602338

**Published:** 2005-01-18

**Authors:** A Mukherjee, A D Westwell, T D Bradshaw, M F G Stevens, J Carmichael, S G Martin

**Affiliations:** 1 Department of Clinical Oncology, City Hospital, University of Nottingham, Nottingham NG5 1PB, UK; 2 School of Pharmacy, Centre for Biomolecular Sciences, University of Nottingham, University Park, Nottingham NG7 2RD, UK

**Keywords:** chemotherapy, hypoxia, thioredoxin, colorectal cancer and antiangiogenesis

## Abstract

AW464 (NSC 706704) is a novel benzothiazole substituted quinol compound active against colon, renal and certain breast cancer cell lines. NCI COMPARE analysis indicates possible interaction with thioredoxin/thioredoxin reductase, which is upregulated under hypoxia. Through activity on HIF1*α*, VEGF levels are regulated and angiogenesis controlled. A thioredoxin inhibitor could therefore exhibit enhanced hypoxic toxicity and indirect antiangiogenic effects. *In vitro* experiments were performed on colorectal and breast cancer cell lines under both normoxic and hypoxic conditions and results compared against those obtained with normal cell lines, fibroblasts and keratinocytes. Antiangiogenic effects were studied using both large and microvessel cells. Indirect antiangiogenic effects (production of angiogenic growth factors) were studied via ELISA. We show that AW464 exerts antiproliferative effects on tumour cell lines as well as endothelial cells with an IC_50_ of ∼0.5 *μ*M. Fibroblasts are however resistant. Proliferating, rather than quiescent, endothelial cells are sensitive to the drug indicating potential antiangiogenic rather than antivascular action. Endothelial differentiation is also inhibited *in vitro*. Hypoxia (1% O_2_ for 48 h) sensitises colorectal cells to lower drug concentrations, and in HT29s greater inhibition of VEGF is observed under such conditions. In contrast, bFGF levels are unaffected, suggesting possible involvement of HIF1*α*. Thus, AW464 is a promising chemotherapeutic drug that may have enhanced potency under hypoxic conditions and also additional antiangiogenic activity.

Colorectal cancer is the fourth most common cancer in the world. Surgery is the mainstay of treatment of this tumour with chemotherapy being used in the adjuvant and palliative setting. 5-Fluorouracil remains the cornerstone of chemotherapy for colorectal cancer.

Angiogenesis, the growth of new blood vessels from pre-existing ones, is essential for the development of tumours (Folkman, 1990) as well as for invasion and metastasis. So an agent with both antitumour and antiangiogenic activity would be useful for colorectal cancer therapy.

AW464 (NSC 706704) is a novel benzothiazole substituted quinol compound with selective activity concentrated in certain colon (HCT116 and HT29), renal (CAKI-1 and ACHN) and breast cancer cell lines (MCF7, MDA-N and MDA-MB435) on the NCI panel (Wells *et al*, 2003). COMPARE analysis indicates possible interaction with thioredoxin/thioredoxin reductase signalling. The proposed molecular target of AW464, thioredoxin, belongs to a family of small 12 kDa redox proteins that undergo NADPH dependent reduction by the enzyme thioredoxin reductase and in turn reduces oxidised cysteine group on proteins (reviewed by Powis and Montfort, 2001; Hirota *et al*, 2002). AW464 has been proposed to crosslink irreversibly to cysteine residues 32 and 35 of the thioredoxin active site via its two *β*-carbon atoms; the first link is reversible, whereas the second crosslink is thought to be irreversible.

The effects of thioredoxin in the cell are pleiotropic, viz increasing cell proliferation through ribonucleotide reductase (Mau and Powis, 1992), prevention of apoptosis by inhibiting ASK-1 (Saitoh *et al*, 1998) and protection against oxidative stress through the activity of thioredoxin peroxidases (Powis and Montfort, 2001). Therefore, inhibition of thioredoxin can have antiproliferative and proapoptotic effects (Pallis *et al*, 2003). Thioredoxin is upregulated in a wide variety of tumours such as mesothelioma (Kahlos *et al*, 2001), leukaemia (Shao *et al*, 2001), lung (Kakolyris *et al*, 2001), hepatocellular (Miyazaki *et al*, 1998) and renal cancer (Lichtenfels *et al*, 2003). Colorectal cancer tissues have also been shown to overexpress thioredoxin and hence thioredoxin could be an important therapeutic target for colon cancer (Raffel *et al*, 2003). Preliminary immunohistochemical studies suggested that in normal human colon thioredoxin is found in the dividing crypt cells, while in colorectal cancer thioredoxin is overexpressed in cancer cells (Powis *et al*, 1998). Thioredoxin has also been reported to be upregulated during hypoxia (Berggren *et al*, 1996) and this, we hypothesise, may lead to increased AW464 efficacy under hypoxic conditions. Through its activity on HIF1*α*, thioredoxin can also regulate VEGF levels and hence angiogenesis (Welsh *et al*, 2002). A thioredoxin inhibitor such as AW464 may thus have both direct and indirect antiangiogenic effects and enhanced hypoxic toxicity.

This study aimed at studying the *in vitro* effects of AW464 on colorectal cancer and on angiogenesis.

## MATERIALS AND METHODS

### HUVEC isolation

Human umbilical vein endothelial cells (HUVEC) were isolated from umbilical cords obtained from the Department of Obstetrics, City Hospital, Nottingham, by the collagenase perfusion technique (Jaffe *et al*, 1973). Briefly, the umbilical vein was cannulated and 5 ml of prewarmed type 1 Collagenase (Lorne Laboratories, Twyford, UK) was infused through it. The free end of the cord was clamped and then incubated at 37°C in a 5% CO_2_ environment. The vein was flushed with 20 ml of HUVEC medium and the isolated endothelial cells centrifuged at 1500 r.p.m. for 5 min. The supernatant was aspirated and the pellet resuspended in 5 ml of HUVEC medium and plated on a pregelatinised tissue culture flask (Sarstedt, UK). Cells isolated from at least two umbilical cords were pooled for further culture. Cells for experiments were used between passage 2 and 6.

### Tumour and normal cells

The colorectal cancer cell lines HT29, SW480, SW620 and the breast cancer cell line MCF7 (American Type Culture Collection) were chosen for this study. SW620 and SW480 form a matched pair of primary and metastatic population of cells from the same patient.

Several normal cell types, in addition to HUVEC, were used for comparison. These include MRCV lung fibroblasts (ECACC), NHDF (normal human dermal fibroblasts), NHEK (neonatal human epidermal keratinocytes) pooled (Cambrex, UK) and HUMMEC (human mammary microvessel endothelial cells) (a gift from Dr C Murray, University of Nottingham).

HT29 cells were used between passage 140 and 150, SW480 between 110 and 120, SW620 between 100 and 110, MCF7 between passage 20 and 30, MRCV between 22 and 26, NHDF between 3 and 8 and NHEK between 1 and 10. HT29 cells were grown in McCoy's 5a medium, SW620 and SW480 in L-15 medium and MCF7 in RPMI 1640 medium. All media were supplemented with 10% Fe-supplemented donor calf serum (PAA Laboratories, Somerset, UK), 1% penicillin–streptomycin (Sigma, Dorset, UK) and 1% L-glutamine (Sigma). MEM medium for growth of MRCV fibroblasts was additionally supplemented with 1% HEPES buffer (Sigma), 1% nonessential amino acids (Sigma) and 1% sodium bicarbonate (Sigma). NHDF fibroblasts and NHEK keratinocytes were grown in FGM-2 bullet-kit medium (Cambrex) and KGM bullet-kit medium (Cambrex), respectively. HUVEC and HUMMEC were grown in a 1 : 1 mix of Hams F12 medium (Sigma) and Medium 199 (Sigma), sterile water (Baxter, UK) supplemented with 20% Fe supplemented donor calf serum (PAA laboratories, UK), 1% HEPES solution, 1% sodium bicarbonate, 1% penicillin–streptomycin, 1% L-glutamine, 15 000 U of Heparin (CP Pharmaceuticals Limited, Wrexham, UK), 20 ng ml^−1^ bFGF and 5 ng ml^−1^ EGF (Peprotech, UK). All media were stored at 4°C for no more than 1 month.

### Assays

#### MTS assay

Cells were plated on flat-bottomed 96-well plates (Nucleon, Riskily, Denmark) in a volume of 180 *μ*l of the respective culture medium at an appropriate seeding density for each cell line to ensure that cells were still in the exponential phase of growth at the end of the incubation period. These were 1000 cells per well for MCF7, HT29, HUVEC, HUMMEC, MRCV and NHDF and 5000 cells per well for SW620, SW480 and NHEK. For HUVEC, the 96-well plates were pregelatinised with 0.2% gelatin in PBS. After 24 h incubation, 20 *μ*l of 10 × drug concentration were added to a triplicate of wells to achieve the drug concentration in a final volume of 200 *μ*l. In total, 20 *μ*l of medium only or vehicle only (DMSO) were added to controls. After 72 h of incubation, 40 *μ*l of MTS-PES reagent (Promega, Southampton, UK) were added to each well and incubated for a further 3 h for colour development. The incubation time was prolonged to 96 h for SW620 and 120 h for SW480 to ensure at least one doubling of absorbance. The time for development of formazan was also increased for these cell lines to 4 h instead of 3 h. The absorbance was read from the plates at 492 nm on a plate reader. Absorbance levels from drug treated cells and untreated controls were corrected against medium only blank controls. The mean absorbance of drug treated wells was expressed as a percentage of nontreated controls to calculate the percentage proliferation status (Carmichael *et al*, 1987).

#### Growth assays

Proliferation status of cells was also assessed by simple counting of cell number after treatment with drugs (Saunders *et al*, 1997). In total, 10^5^ cells of each cell line were plated out on six-well tissue culture plates (Corning, High Wycombe, UK) in a volume of 3 ml of media. The cells were allowed to attach overnight, and then exposed to drugs for 24, 48 and 72 h. A pair of wells were washed with PBS, trypsinised and counted before drug treatment to give the number of cells before the addition of drug. This was taken as the 0 h time point. Further readings were conducted at 24, 48 and 72 h after the addition of drug. Effects were also compared with drug treatment for 48 h of incubation under normoxic and hypoxic conditions. Hypoxia was achieved in a water-jacketed Thermoforma incubator gassed with nitrogen to achieve 1% O_2_.

The effect of the drug on quiescent as opposed to proliferating endothelium were assessed by seeding HUVEC and HUMMEC in six-well plates at a density of 2 × 10^5^ cells well^−1^ and generating a growth curve. When cell numbers reached a plateau, media was aspirated off and replaced with either fresh media for controls or different drug dilutions of AW464 and treated for a further 24, 48 and 72 h.

#### Cell survival assays

While the MTS assay and the growth kinetic assays measure cell proliferation, cell survival assays measure reproductive integrity. These assays were performed according to the protocol of Liebmann *et al* (1993) with some modifications. Petri dishes (100 mm) (Corning, High Wycombe, UK) were plated with 5 × 10^5^ cells in 10 ml of media or 60-mm Petri dishes were plated with 1.8 × 10^5^ cells in 3.5 ml of media. The cells were allowed to attach for 24 h. Media was aspirated off and the exponentially growing cells were then exposed to drug for 72 h, following which they were trypsinised and plated out for colony formation. Incubation time was 4 weeks for all tumour cell lines except SW620 (3 weeks), 2 weeks for fibroblasts and 10 days for HUVEC. Finally, colonies were fixed with methanol (Fisher Scientific, Loughborough, UK) and stained with 1% crystal violet (Sigma) vide a protocol modified from Freshney (1994). Colonies were counted by eye and confirmation by microscopy carried out as necessary. Any cluster of cells greater than 50 in number was counted as a colony. All survival points were in triplicate and experiments repeated at least twice.

#### Tube formation studies on Matrigel

The method was adapted from Dicker *et al* (2001). Petri dishes (100 mm) were plated with 5 × 10^5^ HUVEC cells in 6 ml of media. The cells were allowed to attach for 24 h. The media was aspirated off and exponentially growing cells were then exposed to 6 ml of the IC_50_ dose of control drugs paclitaxel, fumagillin and AW464 for 72 h. For each drug treated condition or control, 50 000 cells in 200 *μ*l of media were then added to wells of a 24-well plate precoated with Matrigel at room temperature, for 1 h. Cells were then incubated at 37°C and 5% CO_2_ for 48 h. Photographs were taken for assessment of tube formation on Matrigel at 24 h postplating.

#### Indirect antiangiogenic effects

Indirect antiangiogenic effects of AW464 were estimated by effects on the production of angiogenic growth factors by tumour cell lines. Cells were plated onto six-well plates at a density of 1 × 10^5^ cells for untreated controls and 2 × 10^5^ cells for drug treated conditions. After attachment overnight, media was aspirated off and 3 ml of different AW464 dilutions or media only were added to the wells. The cells were incubated for a total of 72 h. For the last 24 or 48 h of the incubation period, cells were exposed either to normoxic or hypoxic (1% O_2_) conditions. The supernatant was centrifuged at 2000 r.p.m. for 5 min to remove debris, aliquoted out and stored at −80°C until further use. ELISAs for VEGF and bFGF were conducted according to R&D systems duo-set protocols (Ishibashi *et al*, 2001). Plates were read on a plate reader at 405 nm. VEGF and bFGF production per cell was calculated from the standard curves obtained.

### Statistical analysis

A Student's *t*-test was used to calculate probability values and *P*<0.05 was considered to be statistically significant (indicated by ^*^ on graphs).

## RESULTS

### Cytotoxic effect of AW464 on tumour and endothelial cells

**Figure 1:**
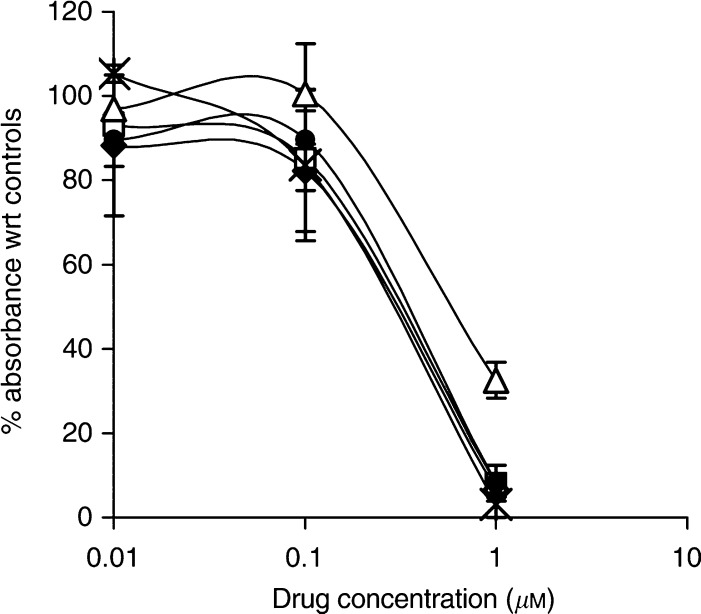
MTS assay results: percentage absorbance as compared to controls after 72 h of treatment with AW464 for HT29 (•), SW480 (▵), SW620 (
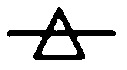
), MCF7 (
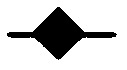
) and HUVEC (^*^) cell lines. Data points were in triplicate in individual experiments and error bars represent standard error of means.

**Figure 2:**
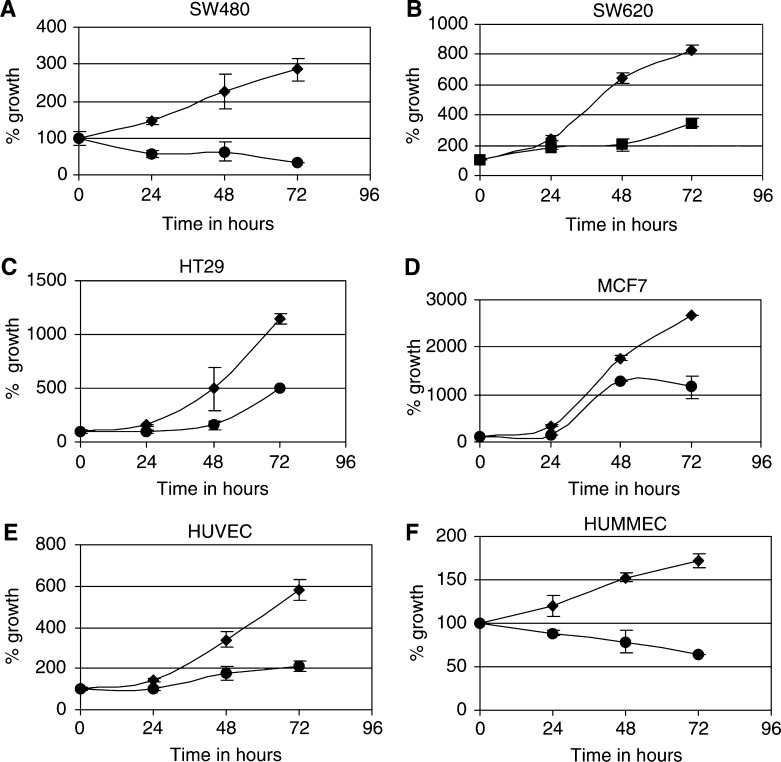
Temporal plots showing action of IC_50_ AW464 (•) and untreated controls (⧫) on SW480 (**A**), SW620 (**B**), HT29 (**C**), MCF7 (**D**), HUVEC (**E**) and HUMMEC (**F**) at 24, 48 and 72 h time points. % growth compared to 0 h time point (time of drug addition) was plotted in duplicate and error bars represent standard deviation.

**Figure 3:**
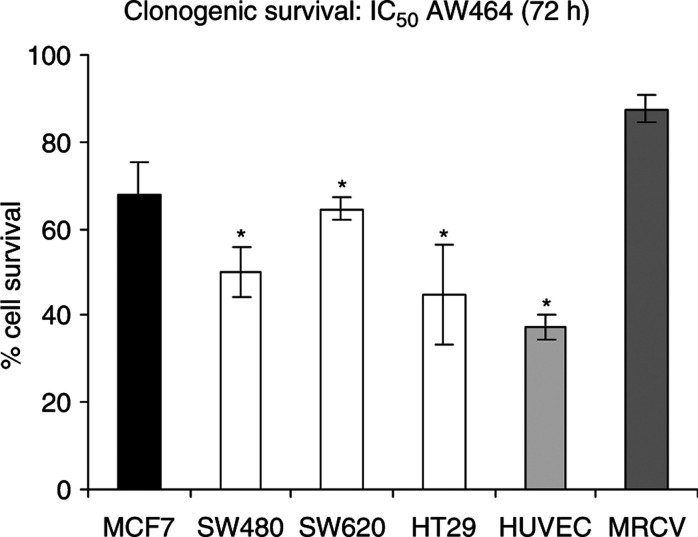
Mean clonogenic cell survival of cells treated with IC_50_ AW464. Data pooled from two experiments with error bars representing standard error of means. Plating efficiencies of individual cell lines with standard error were as follows: MCF7 – 55±5%; SW480 – 20±5%; SW620 – 40±13%; HT29 – 11±0.3%; HUVEC – 22±2%; MRCV – 31±1%.

Both MTS assays (Figure 1) and growth curve experiments (Figure 2) with AW464 demonstrate that the drug inhibits cell proliferation for all tumour cell lines (HT29, SW480, SW620 and MCF7) as well as endothelial cells (HUVEC). The maximum antiproliferative effect is evident after 72 h drug incubation. The IC_50_ (50% growth inhibitory concentration) lies between 0.1 and 1 *μ*M. Cell survival assays, conducted subsequently at the IC_50_ dose (0.5 *μ*M), showed a decrease in clonogenic survival indicating a cytotoxic rather than cytostatic mechanism of action. This profile of action of AW464 was quite similar to the control drug paclitaxel, which also showed a cytotoxic effect on all tumour cell lines as well as endothelial cells (data not shown). MRCV fibroblasts, however, exhibited a relative resistance to the drug (Figure 3). This differential sensitivity was further explored in later experiments (see later).

### AW464 inhibits endothelial tube differentiation

Angiogenesis is a stepwise process where endothelial cells must differentiate into tubes to form new vessels. The effects of the drugs were therefore assessed not only on the proliferation of endothelial cells but also on the ability to differentiate into tubes on a basement membrane matrix, Matrigel. Matrigel is a solubilised basement membrane preparation extracted from the Engelbreth-Holm-Swarm mouse sarcoma. Its major components are laminin, collagen IV and heparan sulphate proteoglycans. HUVEC differentiate into capillary like structures on Matrigel in the presence of serum and growth factors. The formation of tube like vessels on Matrigel can therefore be used to assess compounds that either stimulate or inhibit angiogenesis (Ponce, 2001). AW464 inhibited endothelial differentiation at the IC_50_ dose as evident from tubal abortion at both 24 and 48 h postplating (data not shown). The effects were comparable to the control drugs, paclitaxel and fumagillin.

### Fibroblasts are relatively resistant to AW464

**Figure 4:**
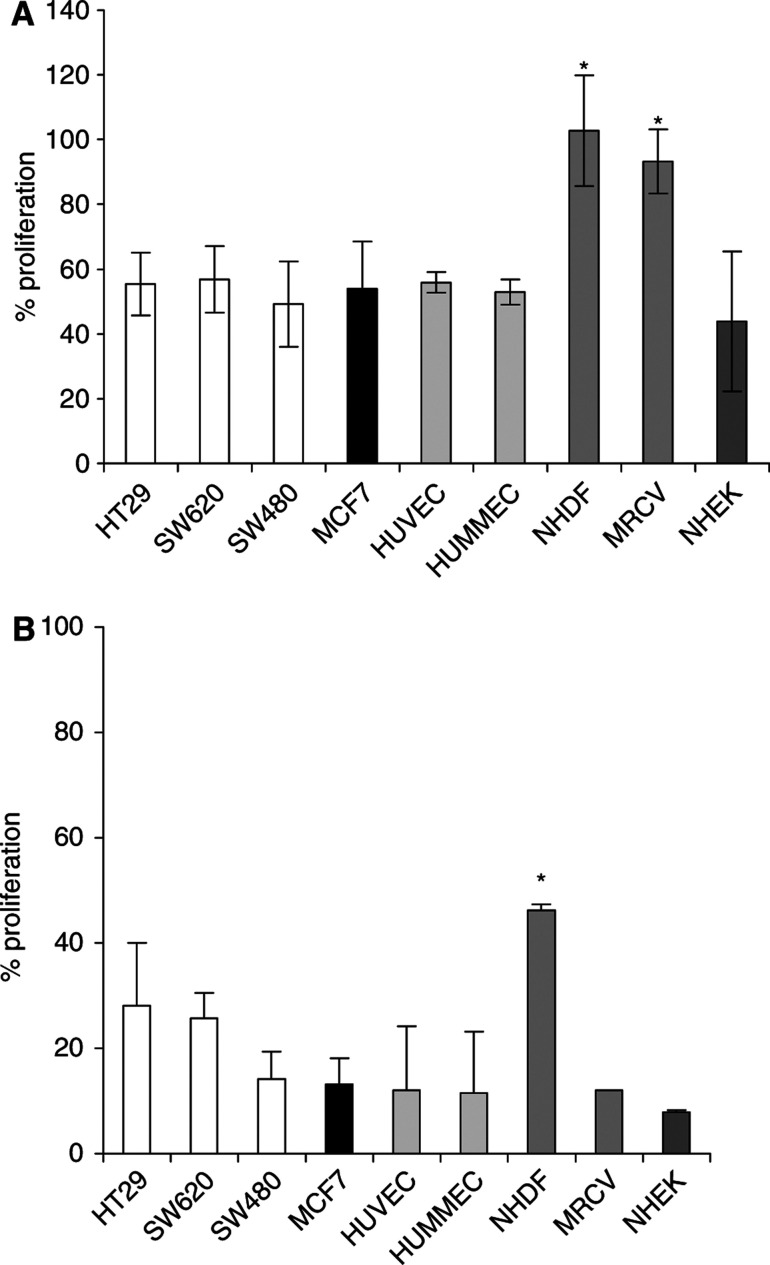
MTS assay results: percentage proliferation as compared to controls after 72 h of treatment with 0.5 *μ*M (IC_50_) (**A**) and 1 *μ*M (**B**) AW464. Data points were in triplicate in individual experiments and error bars represent standard error of means.

The effect of AW464 on the proliferation of different normal cell lines was investigated using the MTS assay (Figure 4). Both large (HUVEC) and microvessel (HUMMEC) endothelial cells and keratinocytes (NHEK) were as sensitive as the tumour lines to the drug with an IC_50_ of ∼0.5 *μ*M. Fibroblasts (NHDF and MRCV) were relatively resistant to the IC_50_ dose (Figure 4A). As the drug dose increases to 1 *μ*M, the MRCV cells become sensitive to the drug but the differential with NHDFs still applies as they are resistant in comparison to the other cell lines (*P*<0.05) (Figure 4B).

### AW464 may be more effective under hypoxic conditions

**Figure 5:**
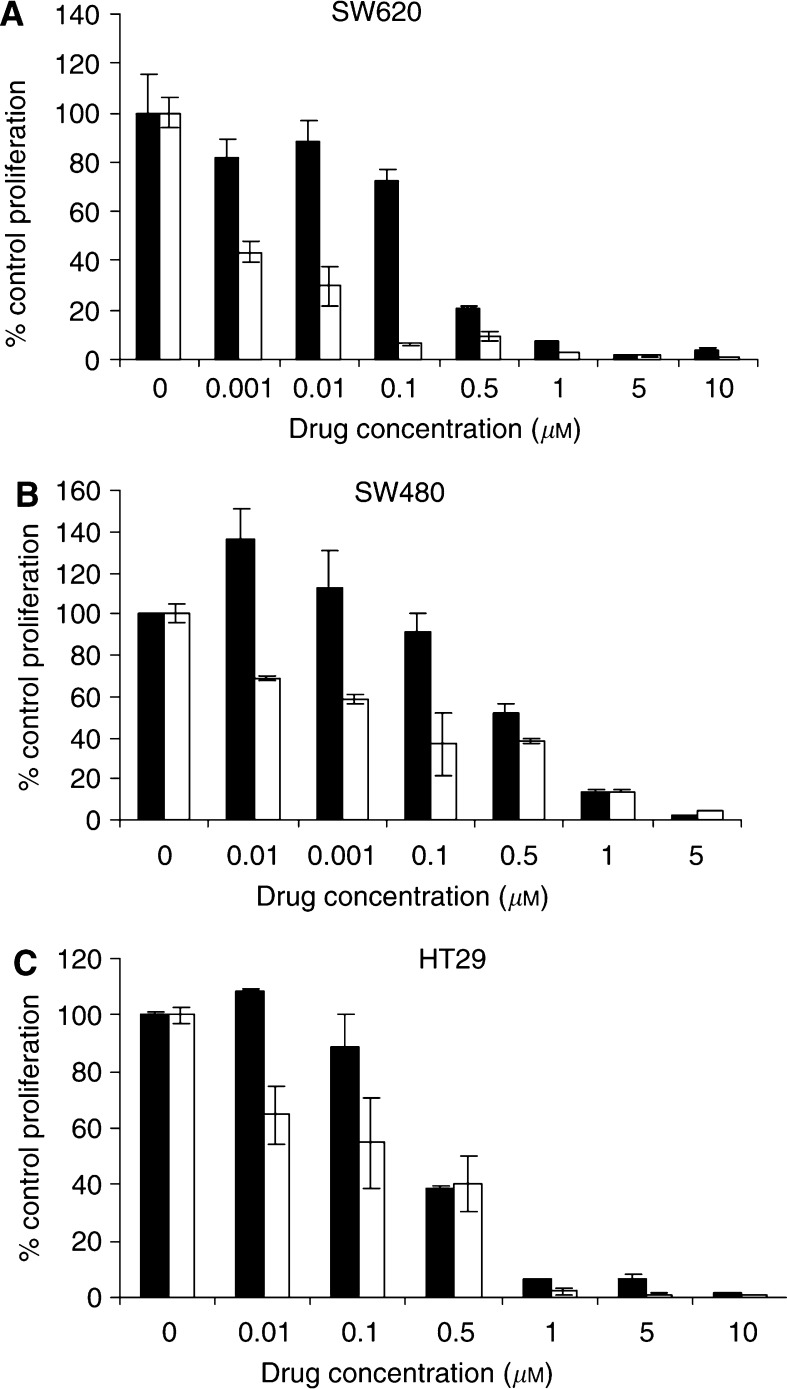
Effects of AW464 on SW620 (**A**), SW480 (**B**) and HT29 (**C**) under normoxia (•) and hypoxia (1% O_2_) (□) for 48 h compared by growth assays. Results were normalised to respective controls with standard deviation depicted as error bars. Mean control cell numbers (× 10^4^ cells) were as follows: SW 620: 31.25 (normoxia); 19.75 (hypoxia); SW480: 37.25 (normoxia); 23 (hypoxia); HT29: 19.87 (normoxia); 17.37 (hypoxia).

As thioredoxin and thioredoxin reductase have been shown to be upregulated under hypoxic conditions (Berggren *et al*, 1996), we investigated whether a potential thioredoxin inhibitor such as AW464 could be more effective under hypoxic conditions. On 48 h of exposure to hypoxia, lower doses of AW464 (0.1–0.001 *μ*M) that had very little effect under normoxic conditions, were effective in decreasing proliferation (Figure 5A–C). There was approximately a five-fold reduction in the IC_50_ (to ∼0.1 *μ*M) for all the colorectal cancer cell lines under hypoxic conditions. These results indicate that AW464 may be more effective under hypoxic conditions.

### Quiescent endothelial cells are resistant to AW464

**Figure 6:**
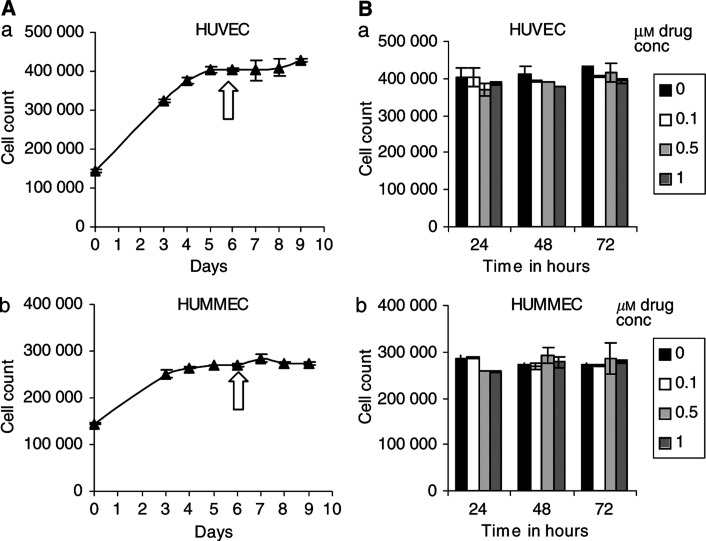
(**A**) (a) HUVEC and HUMMEC (b) were grown to quiescence as indicated by cell numbers on the growth curves. (**B**) At this time-point (arrow), HUVEC (a) and HUMMEC (b) were exposed to different doses of AW464 (no drug control; 0.1; 0.5; 1 *μ*M) and cell numbers were assessed 24, 48 and 72 h later. Readings are from one representative experiment and error bars represent standard deviation.

Antiangiogenic agents should be effective only against proliferating endothelial cells in contrast to antivascular agents that cause vascular shutdown. In order to investigate whether the effect of AW464 on endothelial cells was antiangiogenic, we considered the effects of AW464 on quiescent as opposed to proliferating endothelial cells (Figure 6A(a, b)). AW464 was effective against proliferating large (HUVEC) and microvessel (HUMMEC) endothelial cells but quiescent cells were resistant to the drug over the whole dose range (0.1–1 *μ*M) after 24, 48 and 72 h of exposure (Figure 6B(a, b)). Thus, the mechanism of action of the drug on endothelial cells is potentially antiangiogenic rather than antivascular, although confirmation by *in vivo* experimentation is required.

### Indirect antiangiogenic effects of AW464

**Figure 7:**
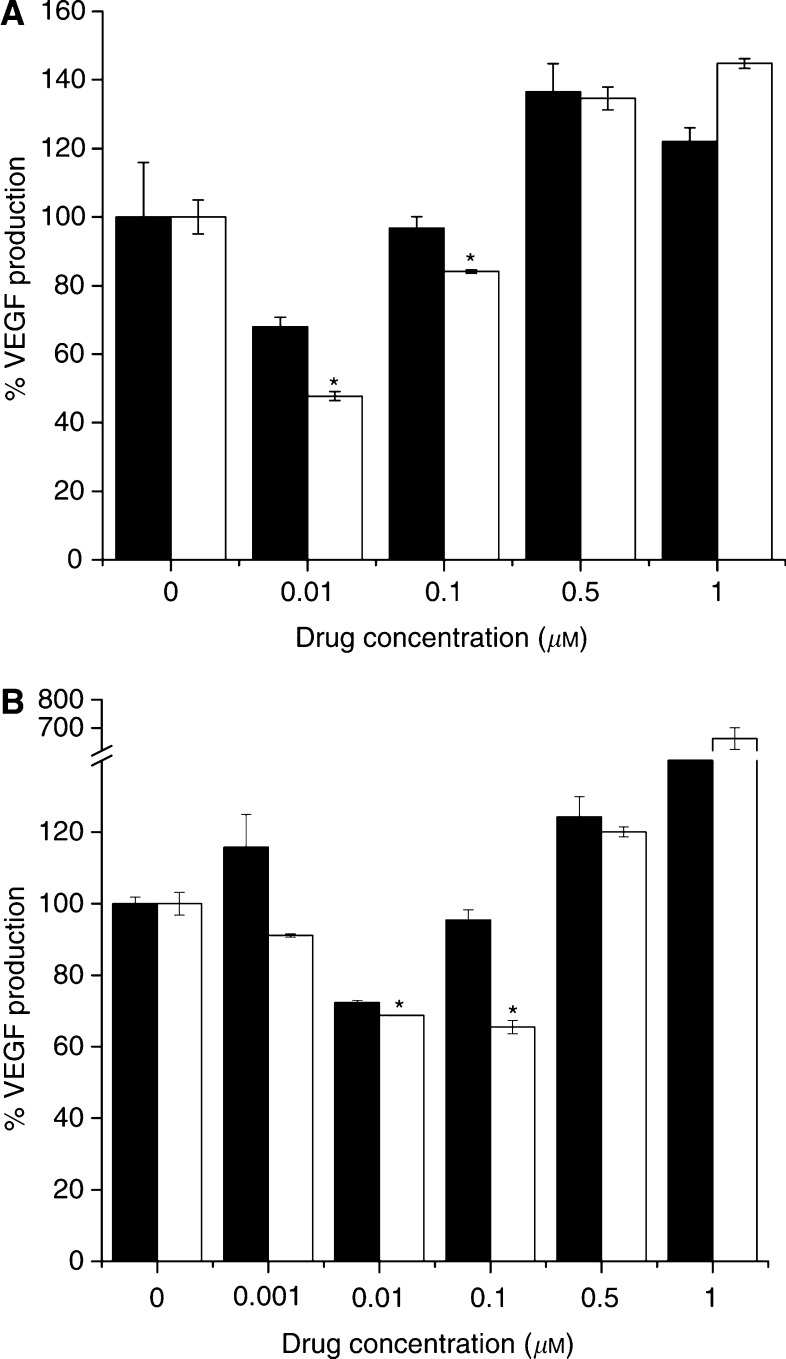
% VEGF production wrt controls at different doses of AW464 under normoxic (▪) and hypoxic (□) conditions in HT29: 24 (**A**) and 48 (**B**) hours of hypoxia. Data from one representative experiment with standard deviation.

Angiogenesis may depend on growth factor production from tumour cells to stimulate the endothelial cells via a paracrine loop. The effect of AW464 on growth factor production was investigated under both normoxic and hypoxic conditions. Low doses of the drug (<IC_50_) cause a decrease in VEGF production (Figure 7A, B). VEGF was upregulated under hypoxic conditions in colorectal cell lines but not by a significant amount. The relative decrease in VEGF levels following AW464 treatment was greater under hypoxic than under normoxic conditions in the HT29 cell line (Figure 7A, B). In contrast, none of the tumour cell lines produced bFGF under normoxic or hypoxic conditions and AW464 had no effects on basal levels (not shown). Since VEGF, unlike bFGF, is under the regulation of HIF1*α*, such results suggest that HIF1*α* may be potentially affected by the drug.

## DISCUSSION

Chemotherapy for colorectal cancer is predominantly used in the adjuvant setting for advanced and metastatic disease. 5-Flourouracil and in recent years, irinotecan, have been the two commonly used drugs for treating this cancer. Unfortunately, median survival for advanced disease is still poor (14.8–17.4 months) (Berlin, 2002). Dukes’ staging remains the most important prognostic factor for colorectal cancer. In the search for new targets for prognosis and survival, several studies have focused on angiogenesis. Some have indicated a positive correlation between angiogenesis and prognosis of colorectal cancer (Frank *et al*, 1995; Pantalone *et al*, 1999), while others have failed to be conclusive (Bossi *et al*, 1995; Lee *et al*, 2000). Among other molecules, thioredoxin has also been shown to be an important prognostic factor for colorectal cancer (Raffel *et al*, 2003) and involved with colorectal carcinogenesis (Berggren *et al*, 1996, Lechner *et al*, 2002). Thioredoxin/thioredoxin reductase signalling therefore may be a novel pathway to target in this malignancy. Thioredoxin and thioredoxin reductase are also important proteins present in endothelial cells to tackle oxidative stress (Fernando *et al*, 1992; Anema *et al*, 1999). So a putative thioredoxin inhibitor may have potential antiangiogenic activity.

The novel drug, AW464, on COMPARE analysis and database mining, was thought to be involved in thioredoxin/thioredoxin reductase signalling. On microarray analysis at the NCI, after treatment of HCT116 cells with 1 *μ*M AW464 for 24 h, the only gene found to be upregulated was thioredoxin reductase (Westwell *et al*, 2003).

In our study, the drug was found to inhibit cell proliferation in colorectal and breast tumour cell lines as well as HUVEC with an IC_50_ of ∼0.5 *μ*M. The antiproliferative effect of a thioredoxin inhibitor may be attributed to the decrease in transfer of reducing equivalents to ribonucleotide reductase from active thioredoxin, leading to decreased DNA synthesis. The effect of the drug at the IC_50_ dose was cytotoxic rather than cytostatic as evident by a decrease in clonogenic survival. In contrast, the proliferation and clonogenic survival of fibroblasts MRCV was unaffected. This led to investigations for relative sensitivities of different cell lines. Both large, HUVEC and microvessel endothelial cells, HUMMEC were found to be as sensitive to the drug as tumour cells. Thioredoxin and thioredoxin reductase have been identified as major redox proteins in HUVEC (Fernando *et al*, 1992; Anema *et al*, 1999) and therefore inhibition of thioredoxin should have direct antiproliferative effects. This was corroborated by this study. The sensitivity of microvessel endothelial cells, HUMMEC, was important as angiogenesis involves the proliferation of microvessel endothelial cells. Inorganic arsenic, another compound that can target the thioredoxin system, has also been shown to be toxic to rat heart microvessel endothelial cells in its trivalent form (Hirano *et al*, 2003) and inhibits endothelial cell proliferation *in vitro* and angiogenesis *in vivo* (Don *et al*, 2003). Both fibroblast cell lines, NHDF and MRCV, are resistant to the IC_50_ dose of AW464. With an increase in dose to 1 *μ*M, fibroblasts also become sensitive to AW464; but in comparison to other cell lines dermal fibroblasts are still relatively resistant. Epidermal keratinocytes, on the other hand, are sensitive. The relative resistance of the fibroblasts cannot be explained by proliferation differences alone and some underlying factor such as differential thioredoxin levels may explain this phenomenon and warrants further investigation. For example, it is known that high levels of thioredoxin are present in epidermal keratinocytes to protect against UV-B-induced skin injury (Schallreuter and Wood, 2001), and this may explain the sensitivity of keratinocytes to AW464. Differential sensitivity assays *in vitro* may be a pointer of drug toxicities *in vivo*. Dermal reactions may therefore be predicted as a toxicity of the drug. Since pulmonary fibroblasts MRCV are relatively resistant, lung toxicities may potentially be minimised.

An antiangiogenic agent should be active only against proliferating and not quiescent endothelial cells, in contrast to antivascular agents. AW464 was found to affect only proliferating endothelial cells, whereas quiescent cells were resistant. The mechanism of action on endothelial cells is thus potentially antiangiogenic rather than antivascular. In addition to there being little effect on normal vasculature there may be significant therapeutic implications in that AW464 may have to be administered chronically or in combination with other agents rather than it being used acutely if it were a tumour vascular targeting agent. Further *in vivo* characterisation is required to confirm whether the drug is a pure antiangiogenic agent or whether it exhibits any antivascular action.

Another therapeutic problem common in tumours is the hypoxic cells at the core that are often resistant to traditional modes of therapy. Thioredoxin expression however is increased under hypoxic conditions (Berggren *et al*, 1996) and hence a thioredoxin inhibitor, we hypothesised, could be more effective under hypoxic conditions. The increased antiproliferative effect of lower doses of AW464 observed under hypoxia may ensure better eradication of the hypoxic population of cells. A low-dose AW464 strategy may thus be combined with radiation or other chemotherapeutic agents that target the oxic cells for greater therapeutic efficacy.

The hypoxic upregulation of thioredoxin also has indirect effects on angiogenesis. Thioredoxin transfection has been shown to increase HIF1*α* transactivation activity and the protein products of hypoxia-responsive genes such as VEGF and nitric-oxide synthase in MCF7 breast and HT29 colon cancer cell lines under both aerobic and hypoxic conditions (Welsh *et al*, 2002). In the current study, low doses of AW464 (<IC_50_) were found to inhibit the production of VEGF and such inhibition was greater under hypoxic conditions. Thioredoxin is known to be upregulated under hypoxia and transfers reducing equivalents to the dual function DNA repair endonuclease Ref1 and thence to HIF1*α* (Ema *et al*, 1999). It then dimerises with HIF1*β* to activate downstream factors such as VEGF. Thus, a thioredoxin inhibitor such as AW464 may stop the transfer of sulphhydryl moieties to HIF1*α* and thereby downregulate VEGF more effectively, as seen in this study, under hypoxic conditions. The colorectal cancer cell lines in this study produced low levels of angiogenic growth factors. AW464 was efficacious in lowering these levels even further, and this indicates that the effect of the drug may be more marked for cell lines that produce high levels of endogenous growth factors. In contrast, bFGF levels were unaffected by the drug or hypoxia. Unlike the HIF1*α*-VEGF signalling pathway, hypoxia induced bFGF expression is mediated through the JNK signal transduction pathway (Le and Corry, 1999). This differential regulation is an indicator that HIF1*α* may be affected by AW464 and experiments are currently underway to examine this. PX-12 (a thioredoxin inhibitor) (Welsh *et al*, 2003a), and pleurotin and PX478 (thioredoxin reductase inhibitors) (Powis *et al*, 2003; Welsh *et al*, 2003b, 2004) have already been characterised as inhibitors of HIF1*α*. However, these thioredoxin inhibitors target the Cys73 of thioredoxin unlike AW464 that is postulated to target Cys 32 and 35.

The decrease in VEGF production at low doses of AW464 (<IC_50_) under normoxic conditions could probably be explained due to the fact that there are also normoxic pathways regulating HIF1*α*. One of these is the PI3 kinase pathway (Zhong *et al*, 2000; Chun *et al*, 2002). Thioredoxin has been shown to regulate this pathway by inhibiting PTEN, which in turn is a negative regulator of the PI3K pathway (Meuillet *et al*, 2004). Hence, thioredoxin inhibitors such as AW464 might indirectly impact on angiogenesis through pathways affecting HIF1*α* under normoxic and hypoxic conditions. The increase in VEGF production at higher doses of AW464 probably represents a stress response, as high doses of chemotherapy have been known to induce VEGF production. In clinical trials with VEGF receptor tyrosine kinase inhibitors, VEGF serum levels increased in patients treated by high doses (Drevs, 2003). Also, heat shock proteins, hsp70 and hsp105, have been shown to be induced at high doses of AW464 in NCI microarrays. Heat shock proteins stabilise HIF1 by preventing its degradation (Zhou *et al*, 2004) and may be involved in this stress response. It should however be borne in mind that the regulation of VEGF expression is quite complex, occurring at transcriptional, post-transcriptional and translational levels and is not exclusively dependent on HIF1 (reviewed by Xie *et al*, 2004). A thioredoxin inhibitor such as AW464 is most likely to inhibit the HIF1 and its associated signalling pathways (e.g. PI3K and Ras pathways) and future assays (macro/microarrays, transactivation assays and Westerns) have been contemplated to further characterise the inhibition at the molecular level.

The efficacy of low doses of AW464 in decreasing proliferation under hypoxic conditions and also decreasing VEGF levels may be utilised by low-dose scheduling of the drug. Recent studies have shown that frequent administration *in vivo* of low doses of chemotherapeutic drugs (‘metronomic’ dosing) can affect tumour endothelium and inhibit tumour angiogenesis, reducing significant side effects (e.g., myelosuppression) (Browder *et al*, 2000; Gately and Kerbel, 2001; Bocci *et al*, 2002). VEGF is not only a proangiogenic factor but can also cause drug/radiation resistance, inhibit apoptosis, impair drug delivery and act as a tumour cell survival factor (reviewed by Harmey and Bouchier-Hayes, 2002). Therefore, lower doses of AW464 that inhibit VEGF, may abrogate all these harmful effects in addition to indirectly preventing angiogenesis. Targeting VEGF may be an important therapeutic tool in colorectal cancer as shown by recent phase II clinical trial results with bevacizumab, a recombinant monoclonal antibody to VEGF (Kabbinavar *et al*, 2003). Time to progression was longer in patients receiving bevacizumab and 5FU-leucovorin than those receiving 5-FU leucovorin alone (9.2 *vs* 5.2 months) (Berlin, 2002). Thus AW464, with its chemotherapeutic and antiangiogenic potential, may be a worthwhile drug for colorectal cancer therapy. Since the subtoxic doses of the drug are most effective in decreasing proliferation under hypoxic conditions and in reducing VEGF levels, it will probably be used as combination therapy without itself causing serious side effects.

From current results, we conclude that AW464, a novel thioredoxin inhibitor, is an effective drug *in vitro* against tumour cells. The added advantage is that its effects may be potentiated under hypoxic conditions at low doses. Fibroblasts are relatively resistant to AW464 and hence some normal tissues may be differentially spared. Potential antiangiogenic effects of the drug are evident in its specificity for proliferating endothelial cells, inhibition of endothelial tube differentiation and reduction in VEGF levels at low doses. bFGF is however unaffected and hence HIF1*α* inhibition is now being investigated. The drug warrants further experimentation, especially *in vivo*, to prove its antitumour and antiangiogenic effects. While *in vitro* results may not always correlate with *in vivo* effects, initial characterisation of novel agents is often conducted in appropriate *in vitro* models. For example, the NCI screen for antiangiogenic agents uses *in vitro* proliferation, tube differentiation on Matrigel and chemotaxis assays in Boyden chambers; a compound positive for effects on any one of these assays is carried forward for *in vivo* analysis. Some preliminary studies for antitumour effects have been conducted with human renal cell carcinoma RXF 944XL hypernephroma xenografts in nude mice (Wells *et al*, 2003). Treatment with AW464 15 mg kg^−1^ on day 1 and day 8 produced 57% growth retardation; treatments on day 1 and 2 produced a growth inhibition of 73%. In HT29 colon xenograft studies, 14 days post-treatment with AW464, there was a tumour growth delay of 7 days accompanied by 42.5% growth inhibition. Further assays are currently being conducted to validate thioredoxin as the potential drug target and elucidate whether HIF1*α* may be inhibited as a consequence. Thioredoxin motifs are also present in molecules such as the protein disulphide isomerases and the endothelial specific PDIs. Whereas PDIs protect endothelial cells during both normoxia and hypoxia, EndoPDIs are upregulated and protect during hypoxic stress (Sullivan *et al*, 2003). Future investigations of potential antiangiogenic mechanisms may involve effects of AW464 on these redox active molecules. Thioredoxin as a molecular target is upstream of several regulatory processes key to the survival of tumours. A potential thioredoxin inhibitor such as AW464 that targets both the tumour and endothelial cell represents an effective therapy for colorectal cancer.
